# 基于*N*-酰基甘氨酸保留指数系统的代谢物色谱峰对齐方法

**DOI:** 10.3724/SP.J.1123.2023.07015

**Published:** 2024-02-08

**Authors:** Jundi HAO, Yaoyu CHEN, Yanzhen WANG, Na AN, Peirong BAI, Quanfei ZHU, Yuqi FENG

**Affiliations:** 1.武汉大学化学与分子科学学院, 湖北 武汉 430072; 1. College of Chemistry and Molecular Sciences, Wuhan University, Wuhan 430072, China; 2.武汉大学公共卫生学院, 湖北 武汉 430071; 2. School of Public Health, Wuhan University, Wuhan 430071, China; 3.武汉大学免疫与代谢前沿科学中心, 湖北 武汉 430071; 3. Frontier Science Center for Immunology and Metabolism, Wuhan University, Wuhan 430071, China

**Keywords:** 保留指数, 色谱峰对齐, 保留时间漂移, 液相色谱-质谱, retention index (RI), peak alignment, retention time shift, liquid chromatography-mass spectrometry (LC-MS)

## Abstract

色谱峰对齐(peak alignment)是非靶向代谢组学分析中常用的数据处理步骤。色谱峰对齐的目的是整合多批次液相色谱-质谱(LC-MS)分析得到的代谢物数据,从而确保数据的可比性与可靠性。然而,微小的色谱分离条件变化会导致连续分析之间的色谱保留时间(retention time, RT)漂移,进而影响色谱峰对齐的准确性。本文提出了一种基于保留指数系统的色谱峰漂移校正策略(retention index-based chromatographic peak-shift correction, RI-based CPSC),应用于代谢物的保留时间漂移校正和色谱峰对齐。为此,我们合成了一系列*N*-酰基甘氨酸(C2~C23)同系物作为校正物,建立了一套液相色谱保留指数系统。该保留指数系统能够有效修正由流速、洗脱梯度、仪器系统和色谱柱变化所引起的色谱保留时间漂移。利用保留指数系统,我们构建了基于Python的程序(https://github.com/WHU-Fenglab/RI-based-CPSC)来调整原始数据的保留时间,以修正系统的保留时间偏移,随后,应用Joint Aligner算法进行色谱峰对齐。以人粪便样作为测试样,评估了RI-based CPSC策略的色谱峰对齐准确性。以一份时间跨度为157天的长期数据为例,RI-based CPSC策略的应用可以将色谱峰对齐率从15.5%显著提高至80.9%,说明RI-based CPSC策略能够显著提高多批次LC-MS分析的色谱峰对齐准确性。

## Abstract (en)

Peak alignment is a crucial data-processing step in untargeted metabolomics analysis that aims to integrate metabolite data from multiple liquid chromatography-mass spectrometry (LC-MS) batches for enhanced comparability and reliability. However, slight variations in the chromatographic separation conditions can result in retention time (RT) shifts between consecutive analyses, adversely affecting peak alignment accuracy. In this study, we present a retention index (RI)-based chromatographic peak-shift correction (CPSC) strategy to address RT shifts and align chromatographic peaks for metabolomics studies. A series of *N*-acyl glycine homologues (C2-C23) was synthesized as calibrants, and an LC RI system was established. This system effectively corrected RT shifts arising from variations in flow rate, gradient elution, instrument systems, and chromatographic columns. Leveraging the RI system, we successfully adjusted the RT of raw data to mitigate RT shifts and then implemented the Joint Aligner algorithm for peak alignment. We assessed the accuracy of the RI-based CPSC strategy using pooled human fecal samples as a test model. Notably, the application of the RI-based CPSC strategy to a long-term dataset spanning 157 d as an illustration revealed a significant enhancement in peak alignment accuracy from 15.5% to 80.9%, indicating its ability to substantially improve peak-alignment precision in multibatch LC-MS analyses.

代谢组学是生物医学研究的重要工具,通过对生物体的代谢物分子表型进行表征,可以揭示生物体的生理状态、代谢途径的变化、调控机制以及与疾病之间的关联性等^[1⇓⇓-4]^。液相色谱-质谱(LC-MS)具有高灵敏度、高选择性和高覆盖度等优点,是目前代谢组学分析研究的主流平台^[5⇓⇓-8]^。然而,生物基质的复杂性以及实验条件的微小变化,常常导致LC-MS分析数据中的保留时间(retention time, RT)和质谱响应出现漂移或波动,这可能会影响多批次代谢组学分析结果之间的可比性^[9⇓⇓-12]^。因此,在基于LC-MS的非靶向代谢组学工作流程中,色谱峰对齐是一个必不可少的步骤^[13⇓-15]^,其目的是将不同批次LC-MS分析得到的代谢物数据进行整合。准确的色谱峰对齐对于确保不同样品之间代谢物数据的可比性和可靠性非常重要。

目前,已开发了多种峰对齐算法,其中应用较广泛的有Correlation Optimized Warping算法^[16]^、Joint Aligner算法^[17]^和Kernel Estimation算法^[18]^,这些算法可以有效地修正由于柱温变化和生物样品的微小差异引起的RT随机偏移;然而,对于流动相组成、色谱柱和仪器条件变化等引起的RT系统偏移,现有峰对齐算法的修正能力有限^[19⇓-21]^。在大规模代谢组学分析中,由于分析周期长、样本量大,RT系统偏移的影响体现更加显著。在这种情况下,如何有效修正RT的系统偏移,提高色谱峰对齐的准确率,是目前代谢组学分析研究面临的一个重要问题^[22⇓-24]^。

保留指数(retention index, RI)是针对一组参考校正物质而言的化合物相对保留时间值,已被广泛应用于气相色谱^[25⇓-27]^。RI可以将不同色谱分析方法所得的RT归一化,使得不同实验室间RT数据具有可比性,有利于分析物的识别和鉴定^[27⇓⇓⇓⇓⇓⇓⇓-35]^。本工作以一系列*N*-酰基甘氨酸同系物(C2~C23)作为校正物,构建了适用于代谢组学分析的RI系统,以修正多批次LC-MS分析过程中RT的系统偏移。基于此,我们还建立了相应的色谱峰漂移校正模型(RI-based CPSC),以提高大规模代谢组学分析中峰对齐的准确性。

## 1 实验部分

### 1.1 仪器、试剂与材料

Agilent 6546 LC/Q-TOF液相色谱-质谱联用系统(美国安捷伦科技公司),包括Agilent 1290 Infinity Ⅱ LC色谱系统和Agilent 6546 Q-TOF质谱仪;Thermo Fisher Scientific LTQ Orbitrap Elite液相色谱-质谱联用系统(美国赛默飞世尔科技公司),包括UltiMate 3000 UHPLC色谱系统和Orbitrap Fusion Tribrid质谱仪。正构脂肪酸(C3~C23,附表S1, www.chrom-China.com)、分析物标准品(73种,附表S1)、甘氨酸(Gly)以及*N*-乙酰甘氨酸(Gly-C2)购自Sigma-Aldrich公司。氯甲酸甲酯、三乙胺(TEA)、四氢呋喃(THF)、氢氧化钠(NaOH)、乙酸乙酯(EA)、盐酸(HCl)、甲醇(MeOH)和甲酸购自国药集团化学试剂公司。LC-MS级乙腈(ACN)购自武汉弗顿科技有限公司。人粪便样品收集自5名健康男性,-80 ℃储存。所有样本采集均严格按照现行人体研究伦理准则进行,并经武汉大学伦理委员会批准(IRB2022001)。

### 1.2 样品制备

粪便样品的制备:将2.0 mL的MeOH加入到含有100 mg粪便的离心管中,涡旋3 min,超声15 min后,10000 g离心5 min,取上层清液,氮气干燥。最后,使用200 μL ACN/H_2_O(1∶9, v/v)对干燥后的样品进行复溶,进行LC-MS分析。

### 1.3 *N*-酰基甘氨酸校正物的合成

在100 mL圆底烧瓶中加入单个正构脂肪酸标准品(500 mg)和15 mL THF,并置于0 ℃低温反应冷阱内。随后,向圆底烧瓶中逐滴加入TEA(250 μL)和氯甲酸甲酯(250 μL),搅拌反应30 min后,加入10 mL的甘氨酸(200 mg)和NaOH(100 mg)混合水溶液,继续反应30 min。反应结束后,使用旋转蒸发仪去除THF,并滴加2 mol/L HCl水溶液(10 mL)使溶液呈酸性。对于*N*-酰基甘氨酸(C8~C23)产物,加入HCl后有白色沉淀生成,通过过滤、洗涤(3 mL水)和干燥,即可获得白色粉末状固体产物;对于*N*-酰基甘氨酸(C3~C7)产物,分别用EA(20 mL×3)进行萃取,合并萃取液,并旋蒸除去EA,得到白色粉末状固体。将购买与合成所得的*N*-酰基甘氨酸(C2~C23)配制为500 μg/L的ACN溶液,储存于-20 ℃冰箱中。

### 1.4 数据处理

LC-MS数据采集使用MassHunter Workstation Version B.06.01 SP1软件(美国安捷伦科技公司)或Thermo Xcalibur 2.1软件(美国赛默飞世尔科技公司)。使用Proteo Wizard msConvert软件将原始数据转换为mzML格式。数据采集时间点用自行开发的RI-based CPSC程序(https://github.com/WHU-Fenglab/RI-based-CPSC)校正后,用MS-DIAL软件(版本4.70)^[36]^进行峰检测、去卷积以及峰对齐(Joint Aligner算法)处理。峰对齐的标准参数设定为RT误差范围0.1 min,质荷比误差(Δ*m/z*)范围0.005。

## 2 结果与讨论

通过对甘氨酸与一系列正构脂肪酸进行酰胺化反应,合成了一系列*N*-酰基甘氨酸同系物(图1a)。为了评估这些*N*-酰基甘氨酸同系物在LC-MS的分析性能,我们进行了色谱保留时间和质谱响应的测试。如图1b所示,在一个固定的25 min色谱梯度条件下(图1b, 虚线), 22个*N*-酰基甘氨酸同系物的保留时间覆盖了0.8~24 min的时间窗口,相邻同系物的保留时间差约为1 min。此外,质谱分析结果显示,在正、负离子模式下,这些同系物均表现出较强的质谱响应,LC-Q-TOF MS分析的定量限(信噪比为10)分别为5.8~93.6 μg/L(正离子模式)和2.3~11.9 μg/L(负离子模式)。

**图1:**
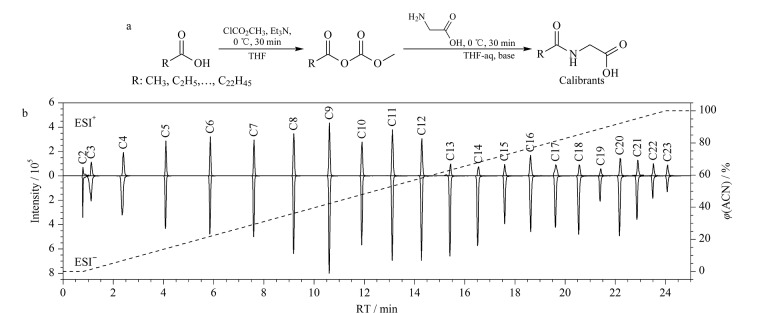
(a) *N*-酰基甘氨酸同系物的合成及(b) 22种*N*-酰基甘氨酸(C2~C23)混合液在LC-MS正、负离子模式下的提取离子流色谱图

以合成的*N*-酰基甘氨酸同系物为校正物,建立了*N*-acyl glycine RI系统。*N*-acyl glycine RI的计算方法如图2a所示。以顺式肉桂醛为例,在正离子模式下,其RT为8.9 min,位于Gly-C7和Gly-C8的保留区间内。通过计算,可得该化合物的RI值为781(图2b(Ⅰ))。类似地,在负离子模式下检测到的3-羟基十二烷酸的RI值也可以被计算为1133(图2b(Ⅱ))。

**图2:**
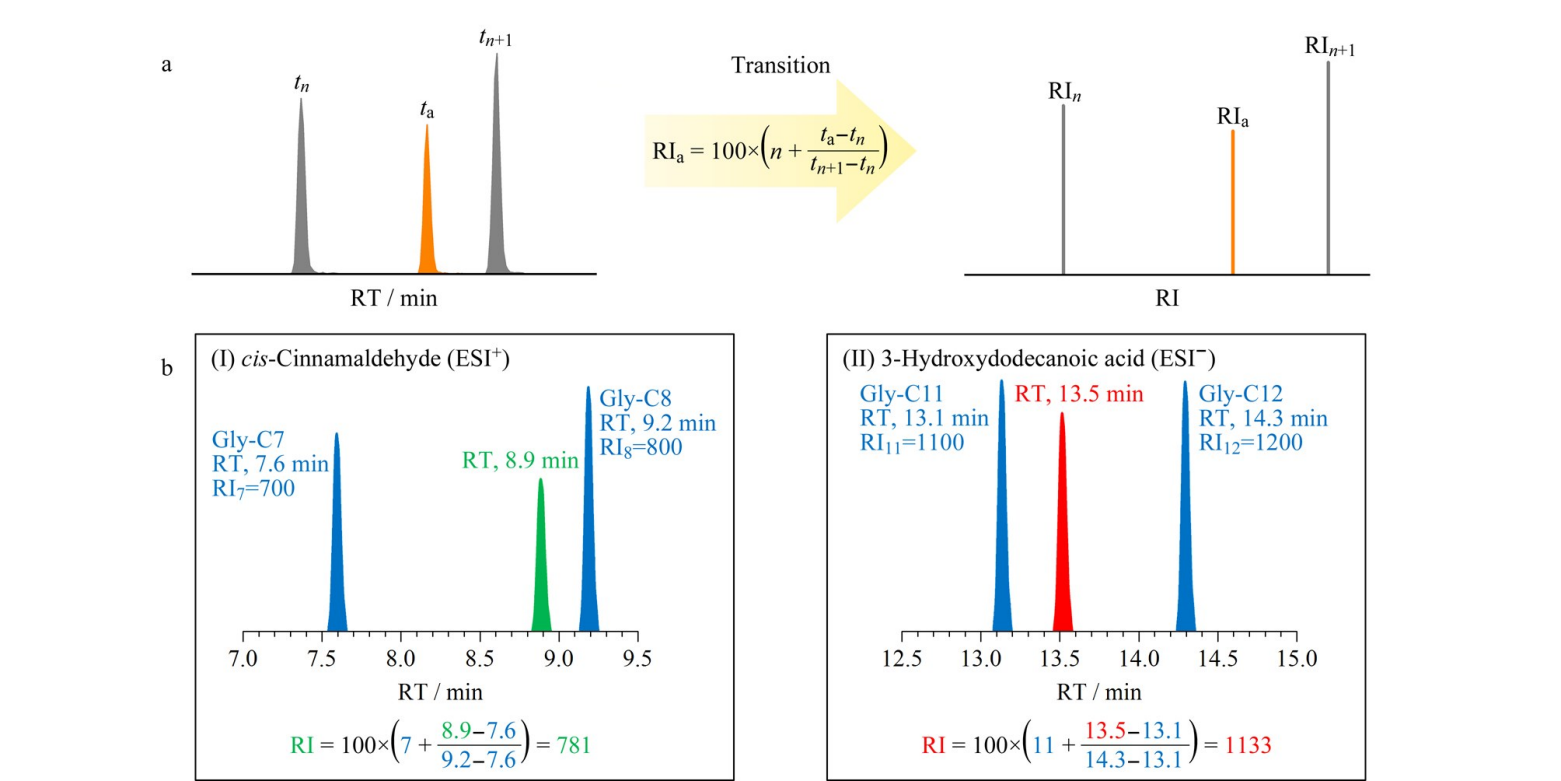
(a)保留指数计算示意图,(b)顺式肉桂醛(Ⅰ)和3-羟基十二烷酸(Ⅱ)的RI计算示例

我们使用了包含73个标准品的混合溶液作为测试样,并考察了在流动相流速、色谱梯度、色谱柱和仪器系统等色谱条件变化的情况下(附表S2),*N*-acyl glycine RI系统对RT系统偏移的校正能力。结果表明,*N*-acyl glycine RI系统可以较好地修正由于上述因素引起的RT偏移(附图S1)。

基于*N*-acyl glycine RI,我们构建了一种新的色谱峰漂移校正模型(RI-based CPCS模型)。该模型使用RI策略对原始LC-MS数据中所有时间点进行修正,使得多次LC-MS分析所得的时间点可与参考样品的时间点对齐,提高了峰对齐准确性(图3)。其中,参考样品指在实际分析过程中,某个被选择作为参考的样品。RI-based CPCS模型的具体工作流程和原理如下。

**图3:**
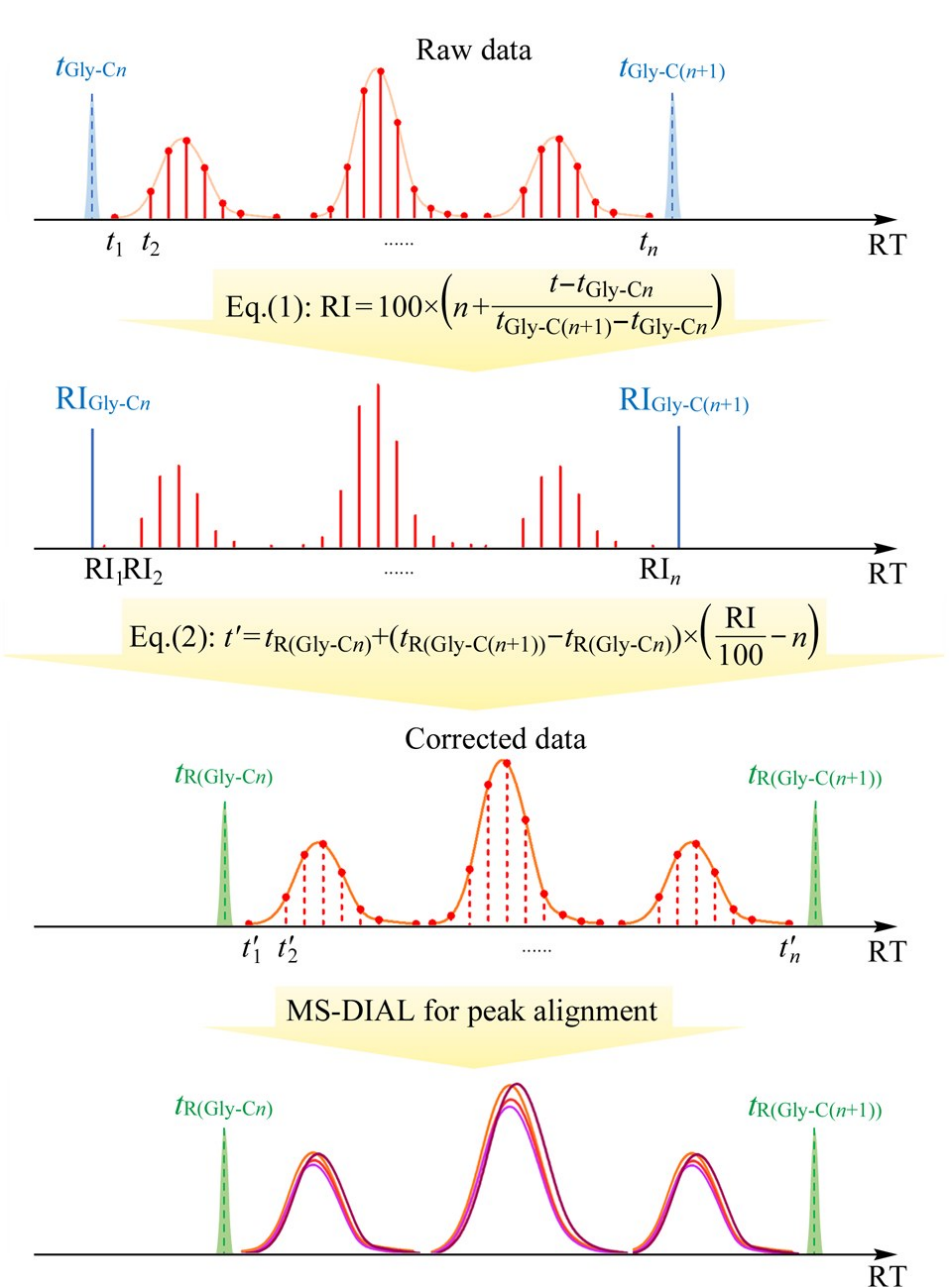
基于*N*-酰基甘氨酸保留指数的色谱峰偏移校正原理图

首先,使用公式(1)计算出色谱时间窗内所有采集时间点所对应的RI值:


(1) $$\mathrm{RI=100\times}\left(n+\frac{t-t_{Gly-Cn}}{t_{Gly-C(n+1)}-t_{Gly-Cn}}\right)$$

其中,*t*_Gly-C_*_n_*和*t*_Gly-C(_*_n_*_+1)_为实际样品中添加的校正物保留时间;*t*为色谱时间窗口内的某一个给定时间点;RI为在该时间点下对应的保留指数;*n*和*n*+1为校正物脂肪酸链的碳数目。

随后,使用公式(2)计算出修正后的时间点,以实现与参考样品的对齐:


(2) $$t^{'}=t_{R\left(Gly-Cn\right)}+\left(t_{R\left(Gly-C\left(n+1\right)\right)}-t_{R\left(Gly-Cn\right)}\right)\times \left(\frac{RI}{100}-n\right)$$

其中,*t*'表示校正后的时间点;*t*_R(Gly-C_*_n_*_)_和*t_R_*_(Gly-C(_*_n_*_+1))_为参考样品中添加的校正物保留时间。

最后,使用开源的代谢组学数据处理软件MS-DIAL对修正后的LC-MS数据进行色谱峰对齐。

为了方便计算,我们还开发了一个基于Python语言的自动化修正程序,该程序可免费获取于 https://github.com/WHU-Fenglab/RI-based-CPSC。

我们对RI-based CPSC模型的峰对齐性能进行了考察。采用同一份人粪便样品作为测试样,分别在第1、49、104和157天进行LC-MS分析。采用随机抽样的方式,共从22 min的LC-MS分析时间窗口内选择220个色谱峰,通过人工检查这些色谱峰在4次LC-MS分析中的峰对齐结果,以评估峰对齐的准确性。结果表明,未经RI-based CPSC模型修正的峰对齐率仅为15.5%;而使用RI-based CPSC模型修正后,峰对齐率则可提高至80.9%。这说明RI-based CPSC模型在处理长时间分析数据时具有显著的优势,能有效提高峰对齐的准确性。该结果也表明RI-based CPSC模型在实际应用中具有可行性和有效性。

## 3 结论

本文提出了一种基于*N*-acyl glycine RI系统的色谱峰对齐新方法。该方法可以实现*N*-酰基甘氨酸校正物所覆盖色谱时间窗内的所有时间点的校正,从而显著提高多批次LC-MS分析所得结果的峰对齐准确度。总之,该方法在大规模代谢组学研究中具有较大的应用潜力。
